# Domestic

**Published:** 1841-04-24

**Authors:** 


### DOMESTIC.

The last number of the American Journal of the Medical Sciences contains another valuable series of statistical tables by Dr. Norris, derived from the practice of the Pennsylvania Hospital. From these tables we learn that the opinion generally entertained of the gravity of certain fractures, is not confirmed by a direct appeal to numbers. Thus, out of nineteen cases of fractured jaw, six died, or nearly one-third; while out of fifteen cases of compound fractured thigh, four were cured and one relieved, or nearly one-third again. These tables afford, therefore, additional proof, if additional proof were necessary, of the necessity of operating upon large masses of cases in order to arrive at just conclusions. They display, also, unfortunately, the loose and general manner of recording cases which, till a very late period, has prevailed in this, as in many other similar institutions.

"The entries being so made in the books of the institution that it is impossible in all cases accurately to ascertain whether the patient was admitted for simple or compound fracture, and for the same reason under the heads of fracture of the arm, and fracture of the leg, are included respectively, those of the fore-arm, either of one or both bones, as well as of the humerus, and of one or both bones of the leg. It is to be regretted that no record of the period required for the union of the different fractures treated, has been kept."

These omissions are not to be attributed to any remissness on the part of the officers, but to the fact that they were not till lately aware of the immense value of a detailed record of cases accurately kept for a succession of years. Had such a record been kept from the commencement of the institution, with the same care which is bestowed upon it at present, what a vast storehouse of facts would now be laid open to us? and what a variety of new truths might be culled from it? How much would the very statistics which we copy have been enhanced in value?

The tables of Dr. Norris, although compiled from books in which this deficiency of detail existed, are however very valuable. They give, as will be seen, statistics of 959 cases of fracture, and 94 cases of dislocation, treated in the Pennsylvania Hospital during 10 years—distributed as follows:

| | *Number.* | *Cured.* | *Relieved or by request.* | *Died.* |
|---|---|---|---|---|
| 1830. | | | | |
| Fractured thigh | 12 | 10 | | 2 |
| Fractured leg | 21 | 20 | | 1 |
| Fractured patella | 3 | 3 | | |
| Fractured cranium | 3 | 2 | | 1 |
| Fractured spine | 1 | | 1 | |
| Fractured ribs | 4 | 4 | | |
| Fractured clavicle | 10 | 7 | 3 | |
| Fractured arm | 27 | 25 | 1 | 1 |
| Fractured fingers | 1 | 1 | | |
| Total, | 82 | 72 | 5 | 5 |
| 1831. | | | | |
| Fractured thigh | 18 | 17 | 1 | |
| Fractured leg | 25 | 22 | | 3 |
| Fractured patella | 2 | 1 | 1 | |
| Fractured cranium | 2 | 1 | | 1 |
| Fractured spine | 2 | | | 2 |
| Fractured ribs | 4 | 3 | 1 | |
| Fractured jaw | 2 | 2 | | |
| Fractured clavicle | 9 | 9 | | |
| Fractured arm | 27 | 25 | 1 | 1 |

| | Number | Cured | Relieved or by request | Died |
|---|---|---|---|---|
| Fractured fingers | 1 | | 1 | |
| Fractured sternum | 2 | 2 | | |
| Fractured scapula | 1 | 1 | | |
| Fractured toes | 1 | 1 | | |
| Compound fractured thigh | 1 | | | 1 |
| Un-united fracture | 1 | | 1 | |
| Total, | 98 | 84 | 6 | 8 |
| **1832.** | | | | |
| Fractured thigh | 8 | 7 | | 1 |
| Fractured leg | 24 | 18 | 1 | 5 |
| Fractured patella | 1 | 1 | | |
| Fractured cranium | 5 | 2 | 1 | 2 |
| Fractured ribs | 3 | 3 | | |
| Fractured clavicle | 9 | 9 | | |
| Fractured arm | 19 | 16 | 2 | 1 |
| Fractured sternum | 1 | 1 | | |
| Fractured pelvis (comp.) | 1 | | | 1 |
| Total, | 71 | 57 | 4 | 10 |
| **1833.** | | | | |
| Fractured thigh | 14 | 12 | | 2 |
| Fractured leg | 31 | 26 | 1 | 4 |
| Fractured patella | 1 | 1 | | |
| Fractured cranium | 8 | 2 | | 6 |
| Fractured ribs | 4 | 2 | | 2 |
| Fractured jaw | 2 | 1 | | 1 |
| Fractured clavicle | 8 | 8 | | |
| Fractured arm | 20 | 18 | 1 | 1 |
| Fractured sternum | 1 | | | 1 |
| Fractured scapula | 1 | 1 | | |
| Fractured nose | 1 | 1 | | |
| Compound fractured thigh | 1 | | | 1 |
| Un-united fractures | 2 | 1 | 1 | |
| Total, | 94 | 73 | 3 | 18 |
| **1834.** | | | | |
| Fractured thigh | 10 | 8 | 1 | 1 |
| Fractured leg | 36 | 31 | 1 | 4 |
| Fractured patella | 3 | 3 | | |
| Fractured cranium | 9 | 4 | 1 | 4 |
| Fractured ribs | 5 | 4 | | 1 |
| Fractured jaw | 2 | 2 | | |
| Fractured clavicle | 10 | 8 | 1 | 1 |
| Fractured arm | 20 | 17 | 2 | 1 |
| Fractured finger | 1 | 1 | | |
| Fractured scapula | 1 | | | 1 |
| Fractured toes | 1 | 1 | | |
| Fracture (?) | 1 | 1 | | |
| Compound fractured thigh | 1 | | | 1 |
| Un-united fracture | 3 | 3 | | |
| Total, | 103 | 83 | 6 | 14 |
| **1835.** | | | | |
| Fractured thigh | 16 | 12 | 1 | 3 |
| Fractured leg | 32 | 26 | | 6 |
| Fractured patella | 4 | 4 | | |
| Fractured cranium | 2 | 1 | | 1 |
| Fractured ribs | 11 | 10 | | 1 |
| Fractured jaw | 8 | 4 | 1 | 3 |
| Fractured clavicle | 9 | 9 | | |
| Fractured arm | 27 | 22 | 3 | 2 |
| Fractured finger | 1 | 1 | | |
| Fractured scapula | 1 | 1 | | |
| Fractured toes | 1 | | 1 | |
| Fractured spine | 1 | | | 1 |
| Fractured pelvis | 1 | 1 | | |
| Compound fractured thigh | 2 | | | 2 |
| Un-united fractures | 2 | | | 2 |
| Fracture | 1 | 1 | | |
| Total, | 119 | 92 | 6 | 21 |
| **1836.** | | | | |
| Fractured thigh | 9 | 8 | 1 | |
| Fractured leg | 27 | 20 | 2 | 5 |
| Fractured cranium | 1 | | | 1 |
| Fractured ribs | 3 | 3 | | |
| Fractured jaw | 3 | 2 | | 1 |
| Fractured clavicle | 8 | 5 | 3 | |
| Fractured arm | 36 | 32 | 2 | 2 |
| Fractured scapula | 1 | 1 | | |
| Fractured spine | 1 | | 1 | |
| Fractured toes | 2 | 2 | | |
| Fractured finger | 2 | 2 | | |
| Compound fractured thigh | 2 | | | 2 |
| Compound fractured knee | 1 | | | 1 |
| Un-united fracture | 1 | 1 | | |
| Total, | 97 | 76 | 9 | 12 |
| **1837.** | | | | |
| Fractured thigh | 6 | 3 | 3 | |
| Fractured leg | 35 | 28 | 2 | 5 |
| Fractured cranium | 5 | 1 | | 4 |
| Fractured ribs | 2 | 2 | | |
| Fractured jaw | 1 | 1 | | |
| Fractured clavicle | 7 | 7 | | |
| Fractured arm | 28 | 23 | 4 | 1 |
| Fractured finger | 1 | 1 | | |
| Fractured spine | 1 | | | 1 |
| Fractured scapula | 1 | 1 | | |
| Fractured nose | 2 | 2 | | |
| Fractured pelvis | 2 | 2 | | |
| Compound fractured thigh | 3 | 2 | | 1 |
| Un-united fracture | 1 | 1 | | |
| Total, | 95 | 74 | 9 | 12 |

| 1838. | Number | Cured | Relieved or by request | Died |
|---|---|---|---|---|
| Fractured thigh | 13 | 9 | 1 | 3 |
| Fractured leg | 34 | 26 | 3 | 5 |
| Fractured cranium | 7 | 1 | | 6 |
| Fractured spine | 1 | | | 1 |
| Fractured ribs | 6 | 5 | 1 | |
| Fractured jaw | 1 | | | 1 |
| Fractured clavicle | 6 | 6 | | |
| Fractured arm | 24 | 20 | 3 | 1 |
| Fractured finger | 2 | 2 | | |
| Fractured scapula | 4 | 3 | 1 | |
| Fractured toes | 2 | 2 | | |
| Fractured os calcis | 1 | | | 1 |
| Fractured knee (comp.) | 1 | 1 | | |
| Un-united fracture | 1 | 1 | | |
| Total, | 103 | 76 | 9 | 18 |

| 1839. | Number | Cured | Relieved or by request | Died |
|---|---|---|---|---|
| Fractured thigh | 12 | 6 | 1 | 5 |
| Fractured leg | 28 | 24 | 1 | 3 |
| Fractured cranium | 4 | | | 4 |
| Fractured ribs | 4 | 3 | | 1 |
| Fractured spine | 1 | | | 1 |
| Fractured sternum | 1 | 1 | | |
| Fractured clavicle | 8 | 7 | | 1 |
| Fractured arm | 22 | 18 | 3 | 1 |
| Fractured patella | 2 | 1 | 1 | |
| Fractured toes | 1 | | 1 | |
| Fractured ilium | 2 | 2 | | |
| Fractured elbow | 2 | 1 | | 1 |
| Fractured foot | 3 | 3 | | |
| Compound fractured thigh | 5 | 2 | 1 | 2 |
| Un-united fractures | 2 | 2 | | |
| Total, | 97 | 70 | 8 | 19 |

*General Summary of the Fractures treated during ten years.*

| Years | Whole number | Cured | Relieved or by request | Died | No. amp. |
|---|---|---|---|---|---|
| 1830 | 82 | 72 | 4 | 5 | 1 |
| 1831 | 99 | 85 | 6 | 8 | 2 |
| 1832 | 71 | 57 | 4 | 10 | 3 |
| 1833 | 94 | 73 | 3 | 18 | 5 |
| 1834 | 103 | 83 | 6 | 14 | 1 |
| 1835 | 119 | 92 | 6 | 21 | 2 |
| 1836 | 96 | 76 | 9 | 12 | 4 |
| 1837 | 95 | 74 | 9 | 12 | 1 |
| 1838 | 103 | 76 | 9 | 18 | 4 |
| 1839 | 97 | 70 | 8 | 19 | 8 |
| Total, | 959 | 758 | 64 | 137 | 31 |

The relative frequency of fractures will be seen in the following table.

| | Number | Cured | Relieved or by request | Died | Amputations |
|---|---|---|---|---|---|
| Fractured thigh | 118 | 92 | 9 | 17 | 2 |
| Fractured leg | 283 | 241 | 11 | 41 | 14 |
| Fractured cranium | 46 | 14 | 2 | 30 | |
| Fractured patella | 16 | 14 | 2 | | |
| Fractured spine | 8 | | 1 | 7 | |
| Fractured sternum | 5 | 4 | | 1 | |
| Fractured clavicle | 84 | 75 | 7 | 2 | |
| Fractured arm | 250 | 216 | 22 | 12 | 9 |
| Fractured fingers | 9 | 8 | 1 | | |
| Fractured scapula | 10 | 8 | 1 | 1 | |
| Fractured elbow | 2 | 1 | | 1 | |
| Fractured nose | 3 | 3 | | | |
| Fractured jaw | 19 | 12 | 1 | 6 | |
| Fractured pelvis | 5 | 5 | | | |
| Fractured foot | 12 | 10 | 2 | | 4 |
| Fractured ribs | 46 | 39 | 2 | 5 | |
| Fracture | 2 | 2 | | | |
| Comp. fractured pelvis | 1 | | | 1 | |
| Comp. fractured knee | 2 | 1 | | 1 | 2 |
| Comp. fractured thigh | 15 | 4 | 1 | 10 | |
| | 946 | 749 | 62 | 135 | 31 |
| Un-united fractures | 13 | 9 | 2 | 2 | |
| Total, | 959 | 758 | 64 | 137 | 31 |

Of the thirty-one amputations performed in cases of fracture, twenty-two were cured and nine died. The details of these have already been given in our statistical account of the amputations made in the hospital, during the years comprised in this report, in the numbers of the American Journal for August, 1838, and May, 1840.

No instance of artificial joint followed the treatment for fracture during the ten years included in the report. All the cases observed there during that period having been sent to the institution from distant parts. Of the thirteen cases that were received, nine were cured, two died, one was benefitted, and one left the house a day or two after entrance without undergoing any treatment for it. Of the fractures included in the above tables, many were compound, and many complicated by other serious injuries.

*Table of the Dislocations treated during ten years.*

| 1830. | Number | Cured | Relieved or by requst | Died |
|---|---|---|---|---|
| Dislocated shoulder | 3 | 2 | 1 | |

| | Number | Cured | Relieved or by request | Died |
|---|---|---|---|---|
| 1831. | | | | |
| Dislocated shoulder | 3 | 2 | | 1 |
| Dislocated hip | 4 | 4 | | |
| Dislocated astragalus | 1 | | | 1 |
| Dislocated cervical vertebræ | 1 | | | 1 |
| Dislocation and fracture | 1 | 1 | | |
| 1832. | | | | |
| Dislocated shoulder | 3 | 3 | | |
| Dislocated hip | 2 | 2 | | |
| Dislocated jaw | 1 | 1 | | |
| Comp. dislocated thumb | 1 | | 1 | |
| 1833. | | | | |
| Dislocated shoulder | 4 | 4 | | |
| Dislocated hip | 1 | | 1 | |
| Dislocated wrist | 1 | 1 | | |
| 1834. | | | | |
| Dislocated shoulder | 6 | 6 | | |
| Dislocated hip | 1 | 1 | | |
| Dislocated ankle | 1 | | | 1 |
| Dislocated wrist | 2 | 2 | | |
| Dislocation | 1 | | 1 | |
| 1835. | | | | |
| Dislocated shoulder | 7 | 7 | | |
| Dislocated hip | 2 | 2 | | |
| Dislocated ankle | 1 | 1 | | |
| Dislocated elbow | 3 | 3 | | |
| 1836. | | | | |
| Dislocated shoulder | 6 | 5 | 1 | |
| Dislocated hip | 3 | 1 | 1 | 1 |
| Dislocated elbow | 2 | 2 | | |
| Dislocated clavicle | 1 | 1 | | |
| Dislocation and laceration | 1 | | | 1 |
| 1837. | | | | |
| Dislocated shoulder | 6 | 6 | | |
| Dislocated hip | 1 | | 1 | |
| Dislocated clavicle | 1 | 1 | | |
| Comp. dislocated thumb | 1 | 1 | | |
| 1838. | | | | |
| Dislocated shoulder | 7 | 7 | | |
| Dislocated hip | 2 | 2 | | |
| Dislocated radius | 1 | 1 | | |
| 1839. | | | | |
| Dislocated shoulder | 4 | 3 | 1 | |
| Dislocated hip | 1 | 1 | | |
| Dislocated jaw | 1 | 1 | | |
| Dislocated clavicle | 1 | 1 | | |
| Dislocated elbow | 2 | 2 | | |
| Dislocated wrist | 1 | 1 | | |
| Dislocated finger | 1 | 1 | | |
| Dislocated great toe | 1 | 1 | | |
| Total, | 94 | 80 | 8 | 6 |

*General Summary of the Dislocations treated during ten years.*

| | Number | Cured | Relieved or by request | Died |
|---|---|---|---|---|
| Dislocated shoulder | 49 | 45 | 3 | 1 |
| Dislocated hip | 17 | 13 | 3 | 1 |
| Dislocated astragalus | 1 | | | 1 |
| Dislocated cervical vertebræ | 1 | | | 1 |
| Dislocated jaw | 2 | 2 | | |
| Dislocated ankle | 2 | 1 | | 1 |
| Dislocated elbow | 7 | 7 | | |
| Dislocated wrist | 4 | 4 | | |
| Dislocated clavicle | 3 | 3 | | |
| Dislocated radius | 1 | 1 | | |
| Dislocated finger | 1 | 1 | | |
| Dislocated great toe | 1 | 1 | | |
| Comp. dislocated thumb | 2 | 1 | 1 | |
| Dislocation and laceration | 1 | | | 1 |
| Dislocation and fracture | 1 | 1 | | |
| Dislocation | 1 | | 1 | |
| Total, | 94 | 80 | 8 | 6 |

A valuable statistical account of the fractures treated in the Pennsylvania Hospitial from its foundation, by Dr. Wallace, is to be found in the second number of the first volume of the Examiner.

---

*Case of Retention of Urine, proving fatal from extravasation and sloughing.*—James Bulkley, aged 38 years, a seaman, native of Connecticut, of intemperate habits, was admitted August 14th, with extravasation of urine. The particulars of his case were as follows:—For three months past, he had been at home without employment, and after drinking to excess the last two weeks, he was confined to his bed with vomiting and purging; four days previous to his admission, he was unable to pass his urine, suffering at the same time from severe pain in the lower part of his abdomen and frequent desire to make water, which only flowed in drops. After attempting to relieve him with poultices and flax-seed tea, his wife became alarmed at the swelling and redness that appeared about the genital organs, and called in a physician the day before his reception, who drew off a large quantity of urine, and apprizing her of his dangerous condition, advised sending him to the hospital. For several years he had passed his urine in a small stream, which he attributed to previous gonorrhœa. The expression of his countenance, when admitted, was bad. Respiration hurried and laboured, and his pulse frequent and small; the tongue dry and coated with a yellowish brown fur. The abdomen below the umbilicus was swollen, very tense and tender to the touch. The swelling obviously depended on thickening and infiltration of the subcutaneous tissues as well as distension of the bladder, which from percussion appeared to extend to within a hand's breadth of the umbilicus. The scrotum and penis as well as the pubes participate in the swelling, and are of a deep red and shining appearance. The tension and hardness are most marked on the pubes and scrotum. The prepuce is infiltrated, transparent, and hides the glans. Erysipelatous redness extends upwards over the flanks towards the back. The perineum, though somewhat swollen, is soft and not painful. Attempts to introduce a silver catheter were unsuccessful, but a small gum elastic bougie, without a wire, passed into the bladder easily. The urine at first flowed in drops, but afterwards more freely, till more than a pint was discharged with evident relief to the patient. A deep incision five inches in length was then made on the left side over the pubes to the scrotum, another upon the same side of the scrotum, from where it joins the thigh down to the raphe at its inferior part. These incisions opened into an extensive deposit of urine that had undermined the integuments around the root of the penis, and along the left side of the urethra as far back as the rectum. This being discovered, the incision of the scrotum was continued backward along the perineum to within an inch of the sphincter. Considerable urine flowed from the incisions, and about six or eight ounces of blood. Three or four ligatures were required, and cloths wet in iced water applied for some time, after which emollient poultices were substituted, and an anodyne draught directed with a liberal allowance of wine whey through the night.

No improvement followed. Sloughing progressed, and the urine escaped from the wound. His bowels continued worse, and the pulse over one hundred. Stimulants and anodynes were given freely, and yeast poultices applied to the sloughing parts. He died August 18th.

*Post-mortem Examination.*—The incisions about the pubes were of a foul appearance, and discharged an offensive dark sanies. Pus was infiltrated into the sub-cutaneous cellular and adipose tissues, extending upwards to within a hand's-breadth of the umbilicus, and along Poupart's ligament nearly to its outer extremity; it was most abundant near the pubes, where the tissues were of a dark olive colour, and in a sloughing condition. The intestines were healthy in appearance, and distended with flatus ; there was no effusion in the cavity of the peritoneum. The bladder was empty and contracted. The membranous portion of the urethra was destroyed ; but, anterior to it, the canal was entire, though the skin covering it was undermined from within an inch and a half of the meatus to the root of the penis. A large cavity was found in the right ischio-rectal fossa that communicated with the urinary deposits of the left side by a passage opposite to the membranous portion of the urethra. On the left side of the prostate gland was a small cavity communicating with the general one, and with the urethra, by a distinct opening in its prostatic portion on the same side. The walls of these cavities, with which the urine had been in contact, were of a dark greenish and purple colour, softened and sloughy. The mucous coat of the bladder, at its fundus, appeared to be gone, and the exposed muscular fibres stood out in relief, and were much developed. The tissues of the bladder, generally, were much thickened. The cœcum, and about two feet of the adjoining ileum, as well as the descending colon, were examined, and presented nothing worthy of notice.—*New York Journal of Medicine and Surgery.*

---

*Smallpox.—Scars prevented by the application of Mercurial Plaster.*—Some years ago, M. Serres at La Pitié, introduced the practice of covering the face of patients with smallpox, with the mercurial plaster of Vigo. Other physicians have since employed the same means with success, and lately M. Chomel of Hotel Dieu, Paris, has repeatedly employed it with the same result. The eruption was modified in all those parts of the body to which the plaster was applied—it was not accompanied by the red areola which surrounds the pustules—the face was less swollen and tense than is common. M. Serres has proved by repeated experiments that other local applications, as the diachylon, gum arabic in water, &c., exert no influence on the eruption. The following cases in M. Chomel's practice will further illustrate this important subject.

*Case* 1.—A young girl, æt. 19, entered the ward St. Augustine. She had been vaccinated, but, as she says, unsuccessfully. The eruption was preceded by the ordinary symptoms, lassitude, pain in the back, vomiting, &c., it is semi-confluent. A mask formed of the plaster of Vigo was applied to the face on the second day of the eruption: twenty-four hours afterwards, the patient tore it off; but notwithstanding the short length of time that it was applied, it produced a remarkable effect. In fact, on the neck, the breast, and every other part of the body, the pustules were developed with all their characteristic marks, they were opake, depressed in the centre, and surrounded by a bright red areola. On the face, the progress of the disease was quite different, and instead of pustules there were only accuminated vesicles and solid papulæ. On certain points, where the plaster had not adhered, there were small pustules, but no where else. A simple inspection of this case is enough to convince any one that the mercurial plaster exerted a beneficial and specific local influence, and that the patient will not be pock-marked.

*Case* 2.—In the same ward, is a patient now convalescent, who has been subjected to the same treatment. Desquamation has followed its usual course in every part of the body except the face, where crusts did not form, except at certain points, where pustules had existed and where the plaster had become puckered.

*Case* 3.—In this case, the patient was five and a half months advanced in pregnancy. The application of the plaster checked the progress of the eruption upon the face, where it was only marked by the appearance of small whitish papulæ. Scabbing did not occur, except about the lips and eyelids, where pustules appeared and where the plaster was not applied.—*Ibid., from Gas. des Hôpitaux.*

### HEALTH OF THE CITY.

INTERMENTS *in the City and Liberties of Philadelphia, from the* 10*th to the* 17*th of April,* 1841.

| Diseases. | Adults. | Children. | Diseases. | Adults. | Children. |
|---|---|---|---|---|---|
| Abscess of lungs, | 1 | 0 | *Brought forward,* | 36 | 41 |
| Apoplexy, | 2 | 0 | Mania a Potu, | 1 | 0 |
| Cancer of stomach, | 1 | 0 | Neuralgia, | 1 | 0 |
| Croup, | 0 | 2 | Old age, | 1 | 0 |
| Consumption of the lungs, | 15 | 2 | Palsy, | 1 | 0 |
| Convulsions, | 1 | 5 | Scrofula, | 1 | 0 |
| Coxalgia, | 0 | 1 | Small pox, | 0 | 3 |
| Dropsy, | 0 | 1 | Still-born, | 0 | 4 |
| —— head, | 0 | 3 | Suicide, | 1 | 0 |
| —— uterus, | 0 | 1 | Spitting of blood, | 1 | 0 |
| Debility, | 0 | 4 | Tabes mesenterica, | 0 | 3 |
| Epilepsy, | 1 | 0 | Unknown, | 4 | 0 |
| Erysipelas, | 0 | 2 | | — | — |
| Enlargement of the heart, | 2 | 0 | Total, 98— | 47 | 51 |
| Fever, | 0 | 2 | | | |
| —— congestive, | 1 | 0 | Of the above, there were under 1 year | | 25 |
| —— typhoid, | 3 | 0 | From 1 to 2 | | 5 |
| —— puerperal, | 1 | 0 | 2 to 5 | | 12 |
| Inflammation of the brain, | 0 | 1 | 5 to 10 | | 6 |
| —— bronchi, | 1 | 3 | 10 to 15 | | 1 |
| —— lungs, | 4 | 3 | 15 to 20 | | 2 |
| —— pleura, | 1 | 1 | 20 to 30 | | 13 |
| —— peritonæum, | 1 | 1 | 30 to 40 | | 15 |
| Marasmus, | 0 | 3 | 40 to 50 | | 4 |
| Measles, | 0 | 6 | 50 to 60 | | 4 |
| Mortification, | 1 | 0 | 60 to 70 | | 6 |
| | — | — | 70 to 80 | | 3 |
| | | | 80 to 90 | | 1 |
| *Carried forward,* | 36 | 41 | Total, | | 98 |

*Weekly Report of Interments in the City and County of New York, from the* 3*d to the* 10*th of April,* 1841.—Diseases: Apoplexy, 1; aneurism, 1; abscess, 1; bleeding from lungs, 2; casualties, 4; cancer, 1; consumption, 27; convulsions, 12; croup or hives, 1; delirium tremens, 2; dropsy, 4; dropsy in the head, 3; dropsy in the chest, 2; drowned, 1; erysipelas, 1; fever, 2; do. scarlet, 4; do. typhoid, 3; do. remittent, 2; intemperance, 1; inflammation of throat, 1; do. of brain, 4; do. of chest, 3; do. of lungs, 12; do. of bowels, 6; do. of stomach, 2; jaundice, 1; marasmus, 8; measles, 3; old age, 1; palsy, 1; small pox, 5; unknown, 1; ulcers, 2; whooping cough, 1.

Ages—Of 1 year and under, 14; between 1 and 2, 14; 2 and 5, 20; 5 and 10, 5; 10 and 20, 7; 20 and 30, 20; 30 and 40, 16; 40 and 50, 10; 50 and 60, 9; 60 and 70, 7; 70 and 80, 2; 80 and 90, 1.

39 men—28 women—37 boys—25 girls. Total, 129.

*Weekly Report of Interments in the City and County of New York, from the* 10*th to the* 17*th of April,* 1841.—Apoplexy 3; Aneurism 1; Asphyxy 5; Abscess 1; Bleeding 1; Bleeding from lungs 1; Cholera Morbus 1; Cancer 2; Consumption 29; Convulsions 5; Croup or Hives 1; Delirium tremens 3; Dropsy 1; Dropsy in the head 11; Drowned 2; Dysentery 1; Erysipelas 6; Epilepsy 1; Fever, scarlet 5; do. typhoid 3; do. puerperal 1; do. remittent 2; do. bilious 1; Gout 1; Hip disease 1; Intemperance 1; Inflammation of womb 2; do. of brain 4; do. of liver 1; do. of chest 3; do. of lungs 18; do. of bowels 3; Killed or murdered 1; Marasmus 7; Measles 2; Old age 2; Organic disease of the heart 2; Palsy 2; Scirrhus 1; Sprue 1; Small Pox 6; Scrofula 1; Teething 2; Unknown 1; Worms 1.

Ages—Of 1 year and under 33; between 1 and 2, 20; 2 and 5 19; 5 and 10, 10; 10 and 20, 2; 20 and 30, 28; 30 and 40, 14; 40 and 50, 16; 50 and 60, 10; 60 and 70, 2; 70 and 80, 8; 80 and 90, 4.

44 men—35 women—53 boys—31 girls. Total, 153.

*Blepharoplastic operations for the restoration of the lower Eyelid, by* J. MASON WARREN, M. D.—The well-known difficulty of remedying the everted state of the eyelid following severe burns and other accidents attended by a destruction of the integuments, has caused a great number of operations to be proposed for its relief.

The method of Dzondi, which has been most generally in use, and which consists in making an incision into the cicatrix, and by maintaining the lips of the wound separate and applying stimulating applications, to induce a full growth of granulations, and thus by a broader cicatrix to remedy the deformity, has altogether failed to produce the desired effect, the eversion almost invariably re-appearing on the healing of the wound.

Within a few years, since the introduction of the autoplastic methods for the restoration of lost parts, the transplantation of cutaneous flaps for supporting the remains of the everted eyelid, has been followed by complete success. The comparative novelty of the operation in this country has led to the publication of the following cases.

CASE I. The subject of the first case was a boy, 12 years of age, from Weymouth, Mass.

When an infant, through the carelessness of his nurse, he was dropped into the fire. The consequence was an extensive burn of the left side of the face, and a partial destruction of the lower eyelid. As the wound in the cheek cicatrized, the remains of the lid were completely everted, and the tarsal cartilage with its ciliæ firmly bound down on a level with the lower edge of the orbit.

The effect of this state of things was a constant epiphora, none of the tears apparently following their natural channels, but running over the cheek and exciting much irritation of the integuments. From the long exposed state of the conjunctiva, this texture had become considerably thickened, presenting almost the appearance of epidermis, and the cornea, from the constant exposure to air, owing to the impossibility of closing the lids, presented an opacity which was daily increasing, and threatened the destruction of vision. Under these circumstances the following operation was performed on the 12th of June—Dr. Hooper, Dr. Salisbury, Dr. Dale, and some other medical gentlemen, being present.

An incision, about an inch and a half in length, was made parallel with the commissure of the eyelids, and about two lines below the palpebral margin, and after a careful dissection, the remains of the eyelid were separated from the edge of the orbit. The dissection was then continued upwards between the tarsal cartilage and the conjunctiva, and the connections so far destroyed as to allow the lid to be restored to its natural position. The thickened and diseased subcutaneous cellular membrane, which might interfere with a proper adhesion of the flap to be transplanted, was then completely removed.

By the separation of the edges of the skin, a large oval-shaped wound now presented, and this was to be filled by a portion of skin taken from a neighboring part. To effect this, an incision was commenced from the outer angle of the wound, and carried, in a semicircular direction, over the temple, at which point, under the hair, was the only portion of sound skin which had not suffered from the effects of the burn; an oval flap was here dissected out, about one-third larger in size than was required, and having fully retracted, was twisted round and maintained in its situation by means of a number of points of the interrupted suture, and a slight pressure exercised upon it with a roller bandage. Before terminating the operation the thickened conjunctiva, which formed a considerable projection beneath the lid, so as to prevent its perfect application to the eyeball, was raised up and entirely removed.

The termination of this case was quite successful. At the end of four days the dressings were removed, and the adhesion of the flap was almost complete—a slight suppuration only, at its inner angle, having occurred. The parts were all considerably swollen. At the end of a week, the pedicle which connected the newly transplanted flap to the neighboring parts was divided, and bled freely, showing a free vascular connection. The patient was sufficiently well in a month to return home.

I saw him about three months after the operation, and he gave the following account of himself.

He was able to close the eye perfectly, and the tears had returned to their proper channels. The newly-formed lid seemed to fulfil all its functions, and there was no disposition in it to become everted. On an examination of the cornea, it was found that the opacity had so far disappeared as to be scarcely perceptible. The only circumstance which required a remedy, was a disposition in the new lid to stand out from the eyeball, as if from a swollen state of the conjunctiva; this was remedied by the repeated application of a pencil dipped in sulphuric acid, so as to destroy a narrow strip of the conjunctiva. The transplanted skin at first formed a considerable protrusion, but is gradually settling down to the level of the surrounding integuments.

Case II. The second case was a young lady, 19 years old. The accident which produced the deformity was very similar to the preceding one—she having been allowed to fall into the fire when an infant, and being very badly burned in the face. From this resulted a very extensive cicatrix, affecting nearly the whole skin of the face, and in some parts implicating the subcutaneous textures. The left eyelid was drawn down and everted at its external angle, leaving the eyeball exposed at that point. From the destruction of the integuments of the cheek, the left angle of the mouth was drawn upward in a direction to meet the external angle of the eye—there being about an inch and a half distance between the two. A large, firm band of indurated and thickened integument extended from the forehead perpendicularly across the bridge of the nose. The external edge of the right eye was also slightly drawn downward by a cicatrix, but the cheek of this side having partially escaped the effects of the burn, there was no eversion of the eyelid. The following operation was planned and executed on the 7th of November, in the presence of Dr. Coates of Philadelphia, Dr. Bethune, and Dr. Warren, Sen.

An incision, two inches in length, commencing on the cheek, midway between the eye and upper lip, was carried with a semicircular sweep in a direction upward and outward towards the ear, its convexity being downward. The skin was then dissected up both above and below, so as to relieve the traction of the integuments in either direction, and on this being accomplished, no difficulty was experienced in restoring the eyelid and angle of the mouth to their natural positions.

From the separation of its lips, the wound on the cheek now gaped widely open, being an inch in the perpendicular, and two inches in the transverse diameter, and this was to be filled up by borrowed integuments. The effects of the burn having penetrated into the muscular substance, it was necessary first to remove all the indurated substance covering the floor of the wound, which was not done without considerable suffering to the patient, this new-formed texture possessing, apparently, a high degree of sensibility. A large oval-shaped flap, one-third larger than was absolutely necessary to fill the wound, was now dissected from the temple, twisted round, and without difficulty adjusted, and secured in its new situation by means of sutures, as in the preceding case. The wound on the temple was drawn together by sutures, and in a direction to favor the transplanted skin in remedying the deformity.

The unseemly cicatrix on the bridge of the nose was now completely dissected out. The vessels which were divided during the operation were allowed to bleed until they ceased voluntarily, it being desirable to avoid ligatures. The wound was dressed with graduated compresses and secured by a roller bandage. The patient was requested to keep perfectly quiet, and not to attempt to use her voice. Notwithstanding these injunctions, she was led to talk considerably during the afternoon, which brought on a slight hæmorrhage, not sufficient to cause much trouble at the time, but which, it was subsequently discovered, had partially prevented the union of the transplanted flap.

But little constitutional irritation followed the operation. On the fourth day the bandage was removed, and two thirds of the flap was found to have united; the inner portion towards the nose was raised up by a coagulum of blood, and the union at this point, of course, defeated. The wound on the temple had, in a great measure, united by the first intention. On the sixth day the ligatures were all removed, and the inner portion of the flap, which showed a disposition to slough, was cut away—the wound, at this point, where, fortunately, the support was least required, being allowed to heal by the second intention.

At the end of six weeks the wounds had all healed, and the patient returned home, greatly improved by the operation. There seemed to be no disposition in the eyelid to become everted, and its functions were well performed. The mouth was also restored to nearly its natural appearance. The facial expression was greatly improved by the removal of the unsightly band which projected out over the bridge of the nose.—*Boston Med. and Surgical Journal.*

